# Regulation of stem-like cancer cells by glutamine through β-catenin pathway mediated by redox signaling

**DOI:** 10.1186/s12943-017-0623-x

**Published:** 2017-02-28

**Authors:** Jianwei Liao, Pan-Pan Liu, Guoxin Hou, Jiajia Shao, Jing Yang, Kaiyan Liu, Wenhua Lu, Shijun Wen, Yumin Hu, Peng Huang

**Affiliations:** 1 State Key Laboratory of Oncology in South China, Collaborative Innovation Center for Cancer Medicine, Sun Yat-sen University Cancer Center, Guangzhou, 510060 China; 2 Department of Translational Molecular Pathology, The University of Texas M.D. Anderson Cancer Center, 1515 Holcombe Blvd., Houston, TX 77030 USA; 3 School of Pharmaceutical Sciences, Sun Yat-sen University, 132 Waihuan East Road, Guangzhou, 510006 China

**Keywords:** Glutamine, Side population, Cancer stem cell, L-asparaginase, Sox2, ROS, β-catenin

## Abstract

**Background:**

Cancer stem cells (CSCs) are thought to play an important role in tumor recurrence and drug resistance, and present a major challenge in cancer therapy. The tumor microenvironment such as growth factors, nutrients and oxygen affect CSC generation and proliferation by providing the necessary energy sources and growth signals. The side population (SP) analysis has been used to detect the stem-like cancer cell populations based on their high expression of ABCG2 that exports Hoechst-33342 and certain cytotoxic drugs from the cells. The purpose of this research is to investigate the effect of a main nutrient molecule, glutamine, on SP cells and the possible underlying mechanism(s).

**Methods:**

Biochemical assays and flow cytometric analysis were used to evaluate the effect of glutamine on stem-like side population cells in vitro. Molecular analyses including RNAi interfering, qRT-PCR, and immunoblotting were employed to investigate the molecular signaling in response to glutamine deprivation and its influence on tumor formation capacity in vivo.

**Results:**

We show that glutamine supports the maintenance of the stem cell phenotype by promoting glutathione synthesis and thus maintaining redox balance for SP cells. A deprivation of glutamine in the culture medium significantly reduced the proportion of SP cells. L-asparaginase, an enzyme that catalyzes the hydrolysis of asparagine and glutamine to aspartic acid and glutamate, respectively, mimics the effect of glutamine withdrawal and also diminished the proportion of SP cells. Mechanistically, glutamine deprivation increases intracellular ROS levels, leading to down-regulation of the β-catenin pathway.

**Conclusion:**

Glutamine plays a significant role in maintaining the stemness of cancer cells by a redox-mediated mechanism mediated by β-catenin. Inhibition of glutamine metabolism or deprivation of glutamine by L-asparaginase may be a new strategy to eliminate CSCs and overcome drug resistance.

**Electronic supplementary material:**

The online version of this article (doi:10.1186/s12943-017-0623-x) contains supplementary material, which is available to authorized users.

## Background

Despite major progress made in our understanding of basic cancer biology and new therapeutic targets in the past decades, the clinical outcomes for certain types of cancers such as lung, liver, and pancreatic cancers remain unsatisfactory. Extensive studies have indicated that cancer stem cells (CSCs) may play a key role in tumor initiation and disease recurrence [1–5], but finding effective measure to eradicate CSCs still remain as a major challenge. Recent advancement in high throughput screening technology has enabled the identification of salinomycin as a selective toxic agent against cancer stem cells [6]. Furthermore, the self-renewal properties of cancer stem cells and the signals from their microenvironment may also be used to preferentially target CSCs. Indeed, the critical role of certain cytokines, pH, and oxygen in affecting CSCs proliferation and differentiation have been evaluated [7, 8]. However, the impact of nutrients in the tumor microenvironment on the CSCs remains largely unknown.

Cancer cells demand rapid ATP production to maintain their active cellular processes, require active biosynthesis of macromolecules to support cell division, and need a tightly controlled ROS metabolism to maintain cellular redox balance and cell survival [9]. Glucose and glutamine are the two major nutrients whose metabolism is often altered in cancer cells. The best characterized metabolic shift in tumor cells is the Warburg effect, which refers to the higher aerobic glycolysis observed in the majority of cancer cells compared to normal cells [10]. Notably, recent studies suggest that CSCs seem to have higher glycolytic activity and lower mitochondria respiration compared to the bulk of “regular” cancer cells [11–13]. Glucose in tumor microenvironment induces a reversible increase of stem-like side population cells [11]. The glucose-driven glycolysis also plays key roles in maintaining hematopoietic stem cells (HSCs) and controlling differentiation, and HSCs exhibit high glycolysis and low oxidative phosphorylation associated with decreased mitochondrial mass and mutations of certain mitochondrial gene [14]. Thus, it is not surprising that glucose in the tumor microenvironment plays an important role in the regulation of stem cell (29).

Glucose and glutamine metabolisms are interrelated at multiple levels. Glutamine transport is the rate-limiting step in the activation of mTOR signaling pathway, and this latter event induces glucose uptake through upregulation of the glucose transporter Glut1 [15, 16]. Glucose and glutamine are both precursors of the tricarboxylic acid (TCA) cycle as well as precursors of the lipids production, nucleotides and amino acids synthesis [17]. However, the effect of glutamine on regulation of CSCs is largely unknown. In this study, we used side population (SP) cells as our in vitro model to study the potential impact of glutamine on stem-like cancer cells. Glutamine depletion from the culture medium resulted in a decrease in SP subpopulation in vitro. We also found that the expression of several key stem cell associated markers (i.e. Sox2 and ABCG2) were also down-regulated upon glutamine deprivation by multiple methods. Moreover, glutamine deprivation led to an increase of reactive oxygen species (ROS), which in turn negatively regulated β-catenin pathway to decrease the fraction of SP cells. Finally, we investigated the potential role of glutamine deprivation and L-asparaginase on A549 cells tumorigenicity capacity in vivo.

## Methods

### Chemicals and reagents

Hoechst 33342, verapamil, glutaminase, L-asparaginase, and 3-Amino-1,2,4-triazole (ATZ), 3-(4,5 dimethylthiazol-2-yl)-2,5-diphenyl tetrazolium bromide (MTT), hydroethidine, Rhodamine 123 were purchased from Sigma (St Louis, MO, USA). Rabbit monoclonal anti-Axin2 (D48G4), rabbit monoclonal anti-Survivin (71G4B7), rabbit polyclonal anti-phospho-β-catenin (Ser33/37/Thr41), rabbit monoclonal anti-phospho-Akt (Ser473) antibodies were obtained from Cell Signaling Technology (Danvers, MA, USA). Mouse monoclonal anti-c-Myc (9E10) was purchased from Santa Cruz (Santa Cruz, CA, USA). Rabbit polyclonal anti-CyclinD1 antibody was obtained from GeneTex (San Antonio, TX, USA). Mouse monoclonal anti-β-catenin (C47H1), rabbit monoclonal anti-Sox-2, rabbit monoclonal anti-ABCG2, and mouse monoclonal anti-β-actin antibodies were purchased from Abcam (Cambridge, UK). CM-DCFDA, Lipofetamine RNAiMAX and Opti-MEM were purchased from Invitrogen Life Technologies (Carlsbad, CA, USA).

### Cells and cell cultures

Human non-small cell lung carcinoma (NSCLC) A549 and pancreatic cancer AsPC-1 cells were obtained from the American Type Culture Collection (ATCC) (Rockville, MD, USA) and routinely maintained in RPMI 1640, supplemented with 10% fetal bovine serum (Invitrogen Life Technologies). Glioblastoma cancer stem cell lines GSC11 and GSC23 originally derived from human glioblastoma tissues were maintained in DMEM/F-12 (Hyclone) supplemented with B-27 (Invitrogen), 2 mM glutamine (Mediatech), 20 ng/ml recombinant human epidermal growth factor (EGF; R&D Systems), and 20 ng/ml basic fibroblast growth factor (bFGF; R&D Systems) as described previously [18]. All cell lines were incubated at 37 °C in a humidified atmosphere with 5% CO_2_.

### Measurement of intracellular ATP

Cellular ATP levels were determined using the ATP-based CellTiter-Glo luminescent Cell Viability kit (Promega, Madison, USA) according to the manufacturer’s instructions with the following modifications. Briefly, Cells were plated in triplicate in 96-well plates to allow for attachment overnight, and then the culture was switched to glutamine-free medium or L-asparaginase (L-ASP) was added to the culture for different times to deplete glutamine. The cell samples were then mixed with equal volume of the single-step reagent provided with the ATP-based CellTiter-Glo kit and rocked for 2 min followed by incubation at room temperature for 15 min. Then luminescence levels were measured using a luminescent plate reader (Thermo Fisher Varioskan Flash; Waltham, MA).

### Flow cytometry analysis of reactive oxygen species (ROS) and mitochondrial membrane potential (MMP)

Intracellular ROS (H_2_O_2_) contents were measured by incubating cells with 10 *μ*M CM-DCFDA at 37 °C for 1 h followed by detection using flow cytometry (Beckman Coulter). Intracellular superoxide level was measured by incubating cells with 50 ng/ml Het at 37 °C for 30 min before detection by flow cytometry. MMP was detected after incubation of cells with 1 *μ*M rhodamine-123 for 30 min flowed by flow cytometry analysis.

### Measurement of cellular glutathione

Cellular glutathione (GSH) concentrations were measured using the GSH-Glo Assay kit (Promega, Madison, WI, USA) according to the manufacturer’s protocol with the following modifications. Briefly, cells were seeded in 96-well plates and incubated with either complete medium or glutamine-free medium for 24 h, 48 h, or 72 h. The culture medium was then removed and the cells were lysed with 100 μl reaction buffer provided in the kit. After incubation for 30 min, 100 μl detection buffer was added and incubated for another 15 min at room temperature. GSH contents were measured using a luminescent plate reader, and were normalized by cell numbers.

### Determination of NADP+/NADPH

NADP+, NADPH, and their ratio were measured using the NADP/NADPH Quantitation Colorimetric Kit (BioVision Inc., Milpitas, CA, USA). Briefly, after A549 cells were cultured with or without glutamine/L-Asparaginase for 48–72 h, the cells were washed with cold PBS and then lysed using NADP/NADPH extraction buffer on ice for 10 min. The cell lysates were spun down and the supernatants were used for measurement of NADP+/NADPH using the assay conditions recommended by the manufacturer (BioVision Inc.).

### RNA extraction and quantitative real-time PCR analysis

Total RNA was extracted with TRIZOL (Ambion, Austin, TX, USA) from A549 cells after cultured in RPMI 1640 with or without glutamine, L-asparaginase or H_2_O_2_. cDNA was generated from equal amount of total RNA (1 *μ*g) using Prime Script RT reagent kit with DNA Eraser (Takara Biotechnology, Dalian, Liaoning, China). Specific primers used for the amplification of indicated genes were listed in Additional file 1: Table S1 and S2. Real-time PCR was performed using SYBR Premix Ex Taq II kit (TliRNase H Plus, Takara Biotechnology, Dalian, Liaoning, China) and the CFX96 real-time system (Bio-Rad Laboratories, Hercules, CA, USA). The RT-PCR amplification reaction program consisted of one cycle of 95 °C/30S and 40 cycles of 95 °C/5S → 60 °C/30S. *β*-actin was used as an internal control for normalization.

### Protein extraction and western blot analysis

A modified RIPA buffer (150 mM NaCl, 50 mM Tris, 0.1% SDS, 1% Triton X-100, 0.5% sodium deoxycholate, 1 mM EDTA) with a protease inhibitor cocktail and a phosphatase inhibitor cocktail (Roche, Indianapolis, Indiana, USA) was used for protein isolation. Cells were washed twice with ice-cold PBS and lysed in 100–200 *μ*l RIPA buffer for 30 min. Cell debris was removed by centrifugation at 12,000 rpm for 15 min at 4 °C. The supernatants were collected, and protein concentrations were determined using the BCA Protein Assay Kit (Pierce, Rockford, IL, USA). An equal quantity of proteins from each experimental condition were subjected to electrophoresis in denaturing 10% SDS-polyacrylamide gel, and then transferred to a PVDF membrane, which was probed for p-β-catenin, β-catenin, p-Akt, ABCG2, SOX-2 and β-actin here using as internal control.

### RNA interference assay

Small RNA interference (siRNA) for knockdown of β-catenin expression in A549 cells was performed using Lipofetamine RNAiMAX Reagent. Briefly, 2x10^5^ A549 cells per well were plated in six-well plates. After overnight incubation, the culture medium in each well was replaced with 2 ml fresh medium containing 250 μl transfection reagents (containing Opti-MEM, β-catenin siRNA or scrambled RNA, and Lipofetamine RNAiMAX). After incubation for 48 h, the transiently transfected cells were collected and RNA was extracted for analysis by qRT-PCR.

### Side population analysis

Cells were washed with PBS, trypsinized, and re-suspended in pre-warmed RPMI 1640 medium containing 2% FBS with or without glutamine at a final density of 1x10^6^ cells/ml. Cell staining was performed according to the method described by Goodell et al. [19] with the following modifications. Briefly, the cells were incubated with Hoechst 33342 (5 *μ*g/ml) in the presence or absence of the ABC transporter inhibitor verapamil (50 *μ*M) for 90 min at 37 °C in dark with intermittent shaking. The cells were then washed and re-suspended in cold PBS. Single cell suspension was obtained using a 70 *μ*m cell strainer. Cells were kept at 4 °C for flow cytometry analysis or sorting on a MoFlo XDP Cell Sorter (Beckman Coulter).

### Colony formation and tumor cell sphere forming assays

A549 cells were seeded in six-well plates at a density of 400 cells per well and cultured at 37 °C for two weeks. At the end of the incubation, the cells were fixed with 100% methanol and stained with 0.1% (w/v) Crystal Violet, and the colonies were counted. Each measurement was performed in triplicate and the experiments were each performed at least three times. For neurosphere formation assay, glioblastoma stem cells GSC11 and GSC23 were seeded in 6-well plates in a range of 100–1000 cells per well, cultured in the indicated medium with or without glutamine for 2 weeks, and then the cell spheres were examined under a light microscope (Nikon).

### Evaluation of in vivo tumorigenicity

To test the effect of glutamine deprivation on tumor-initiating capacity, A549 cells were treated under glutamine deprived conditions for 5 days in vitro. The cells were then harvested and inoculated subcutaneously into the flanks of athymic nude mice with the indicated cell numbers per injection site. The presence or absence of a visible tumor was evaluated and tumor growth was monitored every 3 days. The mice were sacrificed by the end of two months or when the tumors reached a maximum size of 1,000 mm^3^. Tumor volume was calculated by the formula 0.5 × length × width^2^. All animal experiments were conducted in accordance with the institutional guidelines and approved by the Animal Care and Use Committee of Sun Yat-sen University Cancer Center.

### Statistical analysis

Data were analyzed using GraphPad Prism 5 (GraphPad Software, Inc., La Jolla, CA). Data are presented by error bars (mean +/− SD) from experiments in triplicate unless otherwise noted. A two tailed Student’s *t* test was used to determine the statistical significance of difference between samples.

## Results

### Glutamine deprivation reduced stem-like SP cells

**Fig. 1:**
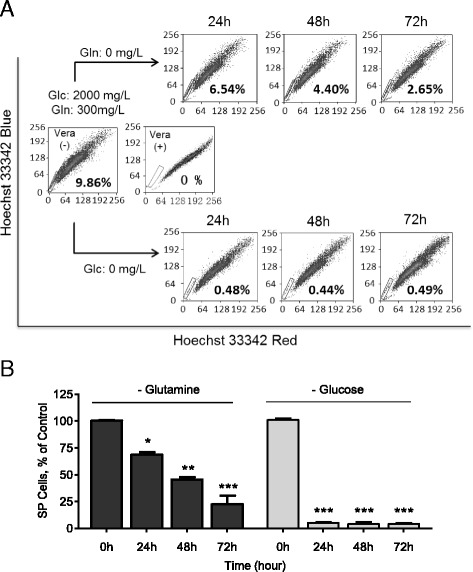
Depletion of glutamine reduced SP subpopulation cells. **a** The human lung cancer A549 cell line was maintained in standard RPMI 1640 medium containing 2000 mg/l glucose and 300 mg/l glutamine. A portion of the cells were switched to glutamine-free RPMI 1640 medium (*upper panels*) and another portion of cells was switched to glucose-free RPMI 1640 medium (*lower panels*). The cells cultured under these different conditions were analyzed for percentage of SP cells at 24 h, 48 h and 72 h. The result of flow cytometry from one representative experiment is shown. **b** Relative quantification of SP fractions under the experiment conditions described in A. Data are means ± SD of 3 independent experiments; *, *p* < 0.05; **, *p* < 0.01; ***, *p* < 0.001. Glc, glucose; Gln, glutamine; Vera, Verapamil

Our previous study has demonstrated that glucose is an important regulator to determine the proportion of side population (SP) in cancer cells through modulating the activity of Akt pathway [11], suggesting that the nutrients in tumor tissue niche may significantly affect the stemness of CSCs. Based on this observation, we further evaluated another important nutrient, glutamine, for its effect on SP cells. Non-small cell lung cancer A549 cells were cultured in RPMI medium with or without glutamine (Gln) for various incubation times and the SP fraction was then analyzed. As shown in Fig. 1a and b, the SP fraction gradually decreased when A549 cells were cultured in Gln-free medium (from 9.86 to 6.54% in 24 h, 4.4% in 48 h, and 2.65% in 72 h). In contrast, glucose deprivation caused a rapid decrease of SP fraction from 9.86% to less than 1% within 24 h (Fig. 1a and b). This significant difference in the time-course of SP decrease suggests that glucose and glutamine might have different mechanisms in regulating SP cells. The impact of glutamine on SP cells was further confirmed in the AsPC-1 pancreatic cancer cell line (Additional file 1: Figure S1).

**Fig. 2:**
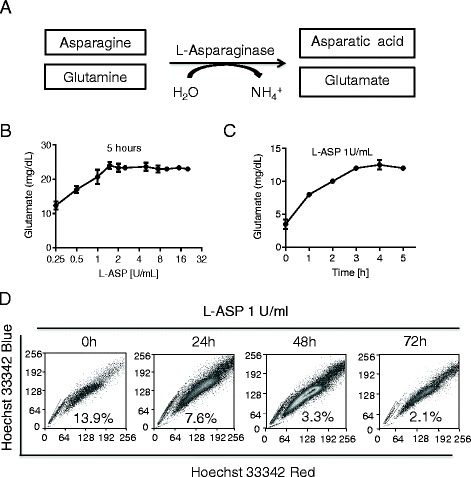
Effect of L-Asparaginase on SP cells. **a** Conversion of asparagine to asparatic acid or glutamine to glutamate catalyzed by asparaginase. **b** Generation of glutamate from glutamine by L-Asparaginase. Cell-free medium containing glutamine (30 mg/dl) was incubated with the indicated concentrations of L-Asparaginase for 5 h, and medium was collected to for measurement of glutamate. **c** Cell-free medium containing glutamine (30 mg/dl) was incubated with 1U/ml L-Asparaginase for indicated time, and the medium was collected for detection of glutamate. **d** A549 Cells were incubated with 1U/ml L-Asparaginase (L-ASP) for the indicated times. Then cells were harvested and stained with Hoechst 33342 to determine SP fraction. The number (%) within each panel indicate the percentage of SP cells in the whole cell population

Based on the above observation that glutamine deprivation significantly affected the fraction of SP cells, we reasoned that blocking glutamine metabolism could also reduce SP cells. For this purpose, a clinical drug L-asparaginase (L-ASP), which catalyzes the hydrolysis of asparagine to aspartate and used in the treatment of acute lymphoblastic leukemia (ALL) in children [20, 21], was used in this study to enzymatically deplete glutamine by its glutaminase activity [22, 23]. As shown in Fig. 2, addition of L-ASP into the cell culture medium caused a concentration- and time-dependent conversion of glutamine to glutamate, and this resulted in a gradual decrease of SP subpopulation (Fig. 2). Consistently, glutaminase also diminished the proportion of SP cells (Additional file 1: Figure S2). These data together suggest that glutamine depletion by either direct removal from the medium or enzymatic depletion significantly diminished the fraction of SP cells.

### Impact of glutamine on clonogenic capacity and expression of stem cell markers

**Fig. 3:**
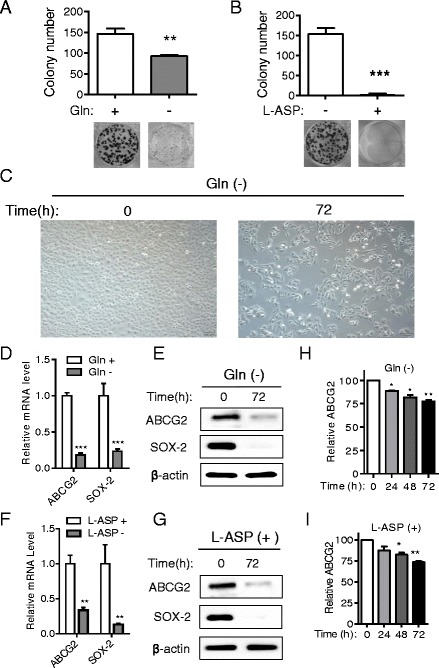
Impact of glutamine on A549 cells clonogenic capacity and the expression of stem-cell associated molecules. **a**-**b** A549 cells were cultured in RPMI 1640 medium with or without glutamine or incubated without or with 1 U/ml L-Asparaginase. Colony numbers were counted after 2 weeks of culturing. The quantitative results of 3 independent experiments are shown in bar graphs showing mean ± SD. Images of representative colonies formed were shown in the lower panels. **c** Representative photographs of A549 cells cultured in medium with or without glutamine for 72 h, original magnification is 400 ×. **d** Effect of glutamine on expression of genes ABCG2 and SOX-2. A549 cells were cultured in RPMI 1640 with or without glutamine (300 mg/L) for 48 h, and RNA was isolated for real-time RT-PCR for detection of SOX-2 and ABCG2 expression. β-actin was used as an internal control for normalization. **e** Western blot analysis of ABCG2 and SOX-2. A549 cells were cultured in RPMI 1640 medium with or without glutamine (300 mg/l) for 72 h, and cell lysates were subjected to western blotting to measure the expression of ABCG2, SOX-2 and β-actin. **f** Effect of L-Asparaginase on expression of genes ABCG2 and SOX-2. A549 cells were cultured in the absence or presence of 1 U/ml L-Asparaginase for 72 h, and RNA was isolated for real-time RT-PCR analysis of SOX-2 and ABCG2. β-actin was used as an internal control. **g** A549 cells were cultured in RPMI 1640 medium without or with L-Asparaginase (1 U/ml) for indicated time, and cell lysates were subjected to western blotting to measure the expression of ABCG2 and SOX-2. (H, I) Effect of glutamine and L-Asparaginase on ABCG2 expression. A549 cells were cultured in RPMI 1640 medium with or without glutamine (**h**) or incubated without or with 1 U/ml L-Asparaginase (**i**) for the indicated time, and cells were collected and incubated with anti-ABCG2 antibody, detection of membrane protein ABCG2 was measured by flow cytometry analysis. Each Bar represents the mean ± SD of the relative fluorescence intensity from 3 independent experiments. *, *p* < 0.05; **, *p* < 0.01; ***, *p* < 0.001; Gln, glutamine; L-ASP, L-Asparaginase

In agreement with the observation that glutamine deprivation or L-ASP treatment reduced SP fractions, both the removal of glutamine and incubation with L-ASP markedly inhibited clonogenic formation in A549 cells (Fig. 3a and b). We also observed that the size of A549 cells became irregular and had flagella-like morphology under glutamine deprivation for 72 h (Fig. 3c). The impact of glutamine on the expression of cancer stem cell markers was further evaluated. As shown in Fig. 3d, the mRNA expression of Sox-2 and ABCG2, two representative markers of stem cells [24–26], was significantly decreased in when glutamine was depleted. Western blot analysis of protein expression (Fig. 3e) further confirmed the results of qRT-PCR analysis. Sox-2 and ABCG2 expression were also decreased after cells were incubated with L-ASP, both at the transcriptional and translational levels (Fig. 3f and g). Since the ABCG2 on the membrane plays a major role in exporting drugs and the Hoechst dye out of the cells, we quantified the change of ABCG2 in A549 cells by flow cytometry in the presence or absence of glutamine or L-asparaginase. ABCG2 expression on the cell membrane was decreased in the absence of glutamine or in the presence of L-asparaginase (Fig. 3h and i). We also tested two glioblastoma stem cell lines GSC11 and GSC23 originally obtained from primary glioblastoma tissues with high levels of stem cell marker CD133 and can easily form neuospheres [12, 27], and showed that glutamine deprivation or L-ASP treatment caused a reduced neurosphere capacity (Additional file 1: Figure S3A and S3B).

To test if the impact of glutamine deprivation on SP cells could be reversed by replenishment of glutamine, A549 cells were first cultured in glutamine-free medium for 48 h to induce a decrease of SP cells. The cells were then switched to glutamine-containing medium for another 48 h, and SP cells were measured. The results showed that there was a substantial recovery of SP population after 48 h in glutamine-replenished medium (Additional file 1: Figure S4A), accompanied by corresponding changes in the expression of stem cell markers including ABCG2, ALDH1, SOX2, and CD44 (Additional file 1: Figure S4B). These data suggest that the effect of glutamine on stemness was reversible.

### Glutamine deprivation increased ROS levels through attenuation of the GSH antioxidant system

**Fig. 4:**
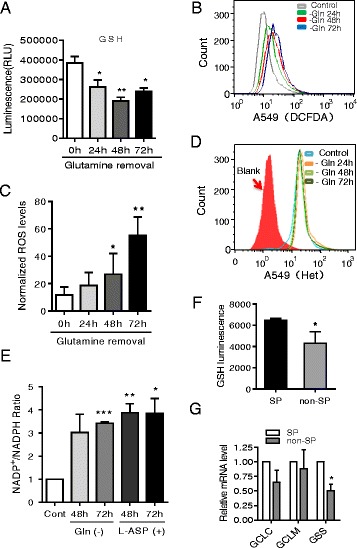
Glutamine depletion leads to a reduction in cellular GSH and ROS accumulation. **a** A549 cells cultured in complete were switched to medium without glutamine for the indicated time. The cells were collected for analysis of intracellular GSH content as described in Materials and Methods. **b** Determination of cellular ROS in A549 cells. Cells were cultured as in (**a**), and ROS was detected by flow cytometry using DCF-DA. **c** Quantitation of cellular ROS under conditions described in (**b**). Each bar represents mean ± SD, *n* = 3. **d** Determination of superoxide in A549 cells cultured with or without glutamine for the indicated time, cellular superoxide was detected by flow cytometry using Het staining. **e** A549 cells were cultured in glutamine-free medium or in complete medium and treated with L-Asparaginase for the indicated time, NADP+/NADPH ratio was then determined as described in Materials and Methods. **f** Comparison of cellular GSH contents in sorted SP and non-SP cells. **g** Comparison of expression of genes involved in synthesis of glutathione in sorted SP and non-SP cells. GCLC, γ-glutamylcysteine ligase catalytic subunit; GCLM, γ-glutamylcysteine ligase modulatory subunit; GSS, glutathione synthetase. Each bar represents the mean of the relative ratio ± SD, *n* = 3; *, *p* < 0.05; **, *p* < 0.01

To investigate the mechanism by which glutamine depletion decreased SP cells, we first tested whether glutamine deprivation could attenuate ATP production, and found that ATP level decreased when glutamine was absent in the culture medium (Additional file 1: Figure S5A), a result similar to that observed with glucose depletion (29). However, unlike glucose depletion which inhibits Akt activation in A549 cells (29), depletion of glutamine did not cause significant decrease in Akt phosphorylation at the time when SP cells were decrease, except a transient decrease at 24 h for a yet unknown reason (Additional file 1: Figure S5B) [11]. This negative result prompted us to further explore other potential mechanisms. Based on the important role glutamine in glutathione (GSH) synthesis and ROS balance that affect stemness of CSCs, we postulated that glutamine deprivation could result in a reduced intracellular GSH content and an increase in ROS accumulation. As shown in Fig. 4a, the absence of glutamine reduced cellular GSH by nearly 40%. As expected, glutamine deprivation also induced a time-dependent increase in intracellular ROS (Fig. 4b and c). However, when we used the superoxide (O_2_^−^) specific probe hydroethidine (Het), we did not observe any change in O_2_^−^ levels under the same experimental conditions (Fig. 4d), suggesting that the increase of ROS was unlikely due to increased O_2_^−^ generation in mitochondria. Indeed, the mitochondrial membrane integrity was not damaged when analyzed by flow cytometry using rhodamine-123 (Rho-123) or nonylacridine orange (NAO) (Additional file 1: Figure S5C and S5D). Moreover, the expression of mitochondrial protein complexes did not change under glutamine free condition (Additional file 1: Figure S5E). We also found that NADP^+^/NADPH ratio increased, consistent with increased ROS caused by GSH depletion (Fig. 4e). These data demonstrated that glutamine deprivation induced glutathione depletion, leading to attenuation of the antioxidant system and an increase of cellular ROS. Consistently, sorting of SP and non-SP cells by flow cytometry revealed that the cellular GSH level was higher in SP cells (Fig. 4f), and the expression of the glutathione synthesis enzyme GSS was higher in the sorted SP cells (Fig. 4g).

**Fig. 5:**
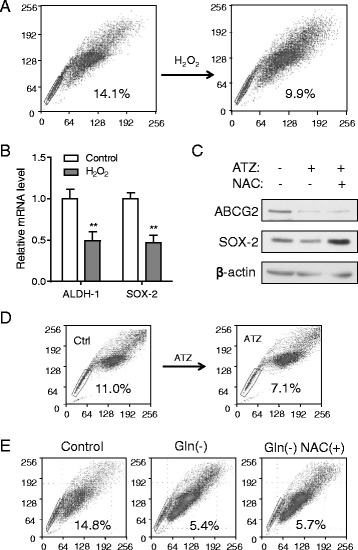
Decrease of SP subpopulation in A549 cells treated with hydrogen peroxide. **a** A549 cells were cultured without or with H_2_O_2_ (50 μM) for 48 h, cells were harvested and stained with Hoechst33342 to determine SP cells. **b** A549 cells were treated without or with H_2_O_2_ (50 μM) for 48 h, then RNA was isolated for real-time RT-PCR for analysis of expression of ALDH-1 and SOX-2. β-actin was used as an internal control. **c** A549 cells was treated with 5 mM aminotriazole (ATZ) for 72 h with or without pretreatment with NAC, cell lysates were subjected to western blotting to measure the expression of ABCG2 and SOX-2. **d** A549 cells were treated with 5 mM aminotriazole (ATZ) for 48 h, cells were harvested and stained with Hoechst 33342 to determine SP cells. **e** A549 cells was treated with glutamine-free medium for 72 h with or without pre-treatment of 2 mM NAC, the cells were collected for SP detection. **, *p* < 0.01

Since previous studies showed that increased ROS levels could induce stem cell differentiation [28–31], we postulated that the effect of glutamine on SP cells could be mediated by change in ROS. Indeed, incubation of A549 cells with 50 μM of hydrogen peroxide (H_2_O_2)_ decreased the proportion of SP cells (Fig. 5a), associated with a reduction in expression of stem cell markers ALDH-1 and Sox-2 (Fig. 5b). The H_2_O_2_-treated cells formed pseudopodia–like morphology (Additional file 1: Figure S6), similar to that observed under glutamine depletion condition (Fig. 3c). Consistently, inhibition of catalase, a key antioxidant enzyme that catalyze the conversion of H_2_O_2_ into water and oxygen [32], by aminotriazole (ATZ) caused a significant decrease in SOX-2 and ABCG2 protein levels, which could be reversed by the antioxidant N-acetyl-L-cysteine (NAC) (Fig. 5c). As expected, ATZ also diminished the percentage of SP cells (Fig. 5d). Interestingly, N-acetyl-L-cysteine (NAC) did not reverse the decrease of side population cells in absence of glutamine in the medium (Fig. 5e), likely due to the inability of cells to use cysteine from NAC for synthesis of glutathione without glutamine (glutathione synthesis requires cysteine, glycine, and glutamine).

### Glutamine regulated the proportion of SP cells through ROS/beta-catenin pathway

**Fig. 6:**
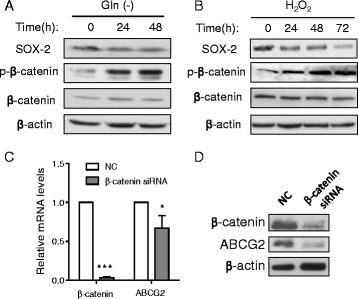
Impact of glutamine deprivation and H_2_O_2_ on β-catenin pathway. **a** A549 cells were cultured in RPMI 1640 medium without glutamine for the indicated time, and cell lysates were subjected to western blotting to measure the expression of SOX2, p-β-catenin, β-catenin, and β-actin. **b** A549 cells were treated 100 μM H_2_O_2_ for the indicated time, and cell lysates were subjected to western blotting to measure the expression of SOX2, p-β-catenin, β-catenin and β-actin. **c** Expression of β-catenin and ABCG2 mRNA in A549 cells transfected with siRNA against β-catenin or with negative control siRNA (NC). *, *p* < 0.05; ***, *p* < 0.001. **d** Expression of β-catenin and ABCG2 protein in A549 cells after siRNA silencing of β-catenin

Considering that Wnt/β-catenin pathway is involved in the maintenance and radiation resistance of cancer stem cells [33, 34] and that ROS could suppress β-catenin pathway through inducing its degradation [35–38], we investigated whether the deprivation of glutamine could exert its effect on SP cells through inhibiting β-catenin pathway. Western blot analysis revealed that depletion of glutamine induced a significant increase in the phosphorylation of beta-catenin, associated with a decrease of SOX-2 (Fig. 6a). Similar results were obtained when the cells were incubated with H_2_O_2_ (Fig. 6b), suggesting that glutamine deprivation and H_2_O_2_ had a similar effect on β-catenin. Consistently, analysis of the β-catenin-regulated genes such as Survivin and Axin2 showed both target molecules were down-regulated at mRNA and protein levels when cells were cultured without glutamine (Additional file 1: Figure S7). However, other β-catenin-regulated molecules (cyclin D1, C-Myc, BCL-2) did not show consistent degrease after glutamine depletion, suggesting that they might also be regulated by other mechanisms.

To further test the role of β-catenin in regulating stem cells, we used siRNA to suppress the expression of beta-catenin, and evaluated its impact on stemness. As shown in Fig. 6c and d, siRNA effectively suppressed the expression of β-catenin, leading to a significant decrease of ABCG2 expression. These data together suggested that glutamine regulated the proportion of stem-like side population cells might be at least in part through ROS-mediated β-catenin phosphorylation, which leads to β-catenin protein degradation by proteasome [38].

### Effect of glutamine on the ability of cancer cells to form tumor in vivo

**Fig. 7:**
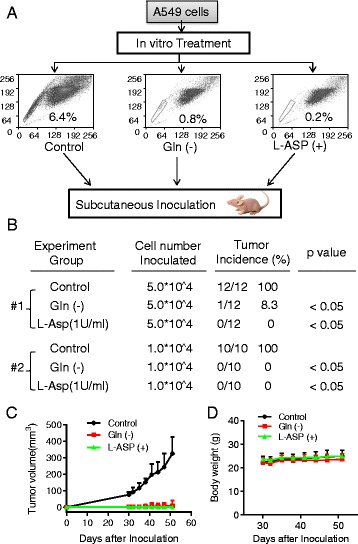
Suppression of tumor formation by glutamine deprivation and L-Asparaginase pretreatment. **a** A549 cells were pre-treated with glutamine-free medium or L-asparaginase (1 U/ml) for 5 days, then equal numbers of viable cells from the control and experimental groups were inoculated subcutaneously into both flanks of athymic mice at the cell number of 5 × 10^4^ cells/injection site (group 1) or 1 × 10^4^cells/injection site (group 2). The mice were then monitored for tumor incidence (**b**) and tumor sizes for group1 (**c**). No tumor formation was observed in cells pre-treated with glutamine-free medium or L-asparaginase in group 2. **d** Body weight curves of mice inoculated with A549 cells as described in A (group 1)

Based on the observations that removal of glutamine or L-ASP incubation could diminish the fraction of SP cells in vitro, we further tested their effect on the ability of A549 cells to form tumor in vivo. As shown in Fig. 7a, A549 cells were first cultured in glutamine-free medium or pre-treated with L-ASP for 5 days, a period of time that is long enough to allow the SP fraction to decrease to less than 1%, as determined by a time-course experiment in which A549 cells were cultured in glutamine-free medium for various times leading to a gradual decrease in SP cells (Additional file 1: Figure S8). The detached dead cells were washed out, and equal numbers of viable cells were inoculated subcutaneously into the flanks of nude mice. Mice in the control and experimental groups (including Gln-free and L-ASP-treated groups) were observed for tumor formation for about 2 months without further treatment. All mice inoculated with 5.0 × 10^4^ control cells developed tumors, while only 1 tumor was observed in the Gln-free group (tumor incidence: 8.3%), and no tumor was found in the L-ASP-treated group (Fig. 7b). When the inoculated cell number was further reduced to 1.0 × 10^4^, the tumor incidence in gln-free or L-ASP-treated group was reduced to 0 while in control group was still 100% (Fig. 7b). Tumor growth was retarded in both Gln-free and L-ASP-treated groups (Fig. 7c). These data demonstrated that glutamine deprivation or L-ASP treatment of A549 cells could severely impair their tumorigenicity in vivo.

## Discussion

Recent studies suggest that the microenvironment in the stem cell niches plays a major role in regulation of stemness, and promotes the long-term survival and self-renew of CSCs [7, 39–42]. Among the nutrients in the tumor microenvironment, glutamine is an important amino acid on which many cancer cells rely for survival and proliferation. In fact, addiction to glutamine is often observed in cancer cells, which use this amino acid as an energy source, a metabolic intermediate for synthesis of other biomolecules, and a precursor for synthesis of glutathione to maintain redox balance [17, 43–45]. Although the reasons for cancer cell dependency on glutamine are not entirely clear, the high demand of energy (ATP) and metabolic intermediates for active cell growth and the increased need for glutathione to counteract ROS stress under oncogenic signals are among the possible explanations for glutamine addiction. Wang et al. revealed that ASCT2 is important for melanoma and inhibition of this glutamine transporter could suppress cell proliferation [46]. In this study, we discovered another important role of glutamine in maintaining stem-like cancer cells, using side population in lung cancer as an experimental model system.

Our study showed that glutamine deprivation induced a significant decrease in SP cells associated with a down regulation of ABCG2 and Sox-2. Interestingly, the rate of decrease in SP cells induced by glutamine depletion was much slower than that induced by glucose deprivation, suggesting that these two major nutrients seem to affect cancer stem cells by different mechanisms. In fact, it has been shown that glucose affects CSCs through a mechanism involving Akt-mediated regulation of ABCG2 expression (29), whereas the current study showed that the Akt activation status seemed not associated with the changes in SP cells induced by glutamine (Additional file 1: Figure S4B). The results of our study suggest that a mechanism by which glutamine affects SP cells may be through ROS-mediated activation of the β-catenin pathway, which regulates the expression of certain stem cell-related genes [47]. Recent studies suggest that CSCs seem to have higher glycolytic activity and may be more dependent on glucose to generate ATP compared to the bulk of general cancer cells [11–13]. Thus, glucose deprivation could cause a severe energy deficiency in CSCs, leading to their rapid decrease. In contrast, a major role of glutamine in CSCs is to function as a metabolic precursor for the synthesis of glutathione to maintain redox balance and keep the intracellular ROS at a relative low level. Thus, a depletion of glutamine would cause an increase of ROS, which tend to induce cell differentiation and eventually lead to a gradual decrease in CSC population. These different roles of glucose and glutamine in energy metabolism and redox homeostasis may explain their different dynamics in impacting CSCs.

In our study, glutamine deprivation caused an increase in β-catenin phosphorylation, leading to its inactivation and a decreased expression of its down-stream targets survivin and Axin2. These results suggest that the β-catenin pathway might play an important role in mediating the down-regulation of SP cells induced by glutamine depletion, which led to an increase in ROS. It is known that ROS negatively regulates β-catenin [36]. Interestingly, a previous study showed that blocking glutamine metabolism could inhibit cancer metastasis [48], which is a property of cancer stem cells. Consistently, we found that glutamine deprivation could induce a decrease of MMP7 (data not shown), a marker of cancer metastasis and also a downstream target of β-catenin.

Cancer stem cells in general are slow-cycling or quiescent cells that retain BrdU-labeling over a long period due to slow division [49, 50], which render them less sensitive to many chemotherapeutic agents that target fast-proliferating cells. A low level of intracellular ROS seems critical to maintain the quiescent status of cancer cells [51]. To maintain a low ROS level, CSCs require high capacity of antioxidants to counteract ROS generated during active cellular metabolism. Indeed, two important transcription factors, FoxO and P53, have been shown to play a significant role in regulation of cellular ROS, and both are considered to be important molecules for the maintenance of stem cells [52, 53]. The reduced form of glutathione (GSH) is a highly abundant antioxidant in the cells, and plays an important role in keeping redox balance and promoting cell viability and drug resistance. In fact, cancer cells with positive CD44, which interact with the cysteine transporter xCT and promote GSH synthesis [54], exhibit grow advantage and resistant to certain therapy [55, 56].

**Fig. 8:**
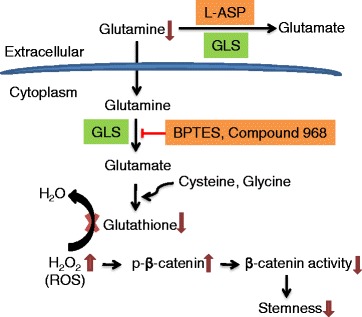
Schematic model for regulation of stem-like side population cells by glutamine. Glutamine is a precursor for glutamine synthesis and is important in maintaining redox balance. Depletion of glutamine would result in a decrease in GSH, and subsequently causes an accumulation of ROS, which in turn induces phosphorylation of β-catenin and thus inactivate this pathway, lead to loss of stemness. Targeting glutamine metabolism either by enzymatic elimination of glutamine in the tumor microenvironment using L-ASP or by inhibition of intracellular conversion of glutamine to glutamate using glutaminase (GLS) inhibitors such as BPTES and compound 968 may decrease cancer stem cells through increasing ROS and attenuation of the beta-catenin activity

The high ability of CSCs to utilize glutamine for GSH synthesis leading to increased cell viability and drug resistance imposes a significant challenge in clinical treatment of cancer. However, our study suggests that the addiction of CSCs to glutamine metabolism could also provide a potential therapeutic target for elimination of CSCs. Furthermore, glutamine is also important to support cancer cells viability and growth through the KRas-regulated metabolic pathway [57]. As such, it seems possible to target glutamine metabolism either by enzymatic elimination of glutamine in the tumor microenvironment using L-ASP or by inhibition of intracellular conversion of glutamine to glutamate using glutaminase (GLS) inhibitors such BPTES and compound 968, as illustrated in Fig. 8. It is worth noting that although direct removal of glutamine from the cell culture medium is a straightforward approach to evaluate the role of glutamine in supporting CSCs in experimental system, it is difficult to deprive glutamine in vivo for therapeutic purpose. However, it may be possible to use enzymes such as glutaminase and L-asparaginase to remove glutamine in vivo to impact cancer stem cells. Since L-ASP is a clinical drug currently used in treatment of ALL largely due to its ability to deplete asparagine and thus suppresses ALL cell proliferation [58], it would be feasible to test the possibility to use L-ASP to eliminate CSCs in a clinical setting. Due to the plasticity of cancer stem cells and possible reversion of downstream cancer cells to stem stage, it may be necessary to combine L-ASP with other anticancer agents to increase the chance to eliminate the entire cancer cell population and achieve better therapeutic outcome.

## Conclusions

Stem–like side population cells are more addicted to glutamine. Deprivation of glutamine can decrease the fraction of SP cells and stem cell markers (SOX-2 and ABCG2). Glutamine deprivation increases cellular ROS through attenuating glutathione synthesis, while increased ROS suppresses β-catenin pathway through inducing its phosphorylation and degradation. Tumor formation capacity in vivo was weakened through blocking glutamine utility by L-asparaginase.

## Additional file

Additional file 1: Figure S1. Effect of glutamine depletion on stem-like SP fraction in pancreatic cancer AsPC-1 cells. **Figure S2**: Effect of glutaminase on percentage of SP cells in A549 cells. **Figure S3**: Effect of glutamine deprivation on neurosphere formation capacity of glioblastoma stem cells. **Figure S4**. Effect of glutamine deprivation and replenishment on stemness of cancer cells. **Figure S5**: Glutamine depletion increases ROS through lowering glutathione level. **Figure S6**: Morphology change induced by hydrogen peroxide. **Figure S7**. Effect of glutamine depletion on the expression of β-catenin targeted genes. **Figure S8**: Effect of glutamine on SP cells. **Table S1**. Primers used for quantitative real-time PCR assay. **Table S2**. Primers used for quantitative real-time PCR assay. (PPTX 762 kb)

## Abbreviations

ABCG2: ATP-Binding Cassette, Sub-Family G (WHITE), member 2
ALL: Acute lymphoblastic leukemia
ASCT2: ASC amino-acid transporter 2
ATZ: Aminotriazole
CSC: Cancer stem cell
FOXO: Forkhead box O
GLC: Glucose
GLN: Glutamine
GSC: Glioblastoma stem cell
GSH: Glutathione
H_2_O_2_: Hydrogen peroxide
Het: Hydroethidine
L-Asp: l-asparaginase
MMP: Mitochondrial membrane potential
MTT: 3-(4,5 dimethylthiazol-2-yl)-2,5-diphenyl tetrazolium bromide
NAC: N-acetyl-L-cysteine
NADP: Nicotinamide adenine dinucleotide phosphate
NAO: Nonylacridine orange
Rho-123: rhodamine-123
ROS: Reactive oxygen species
SOX-2: SRY (sex determining region Y)-box 2
SP: Side population
TCA cycle: Tricarboxylic acid cycle
xCT: x-Cystine-glutamate-trasporter
