# Supplementary material for: Regulation of stem-like cancer cells by glutamine through β-catenin pathway mediated by redox signaling
Source: Mol Cancer. 2017 Feb 28;16:51. doi: 10.1186/s12943-017-0623-x (PMC5331650; doi:10.1186/s12943-017-0623-x)
Supplement: Additional file 1: Figure S1. — Effect of glutamine depletion on stem-like SP fraction in pancreatic cancer AsPC-1 cells. Figure S2: Effect of glutaminase on percentage of SP cells in A549 cells. Figure S3: Effect of glutamine deprivation on neurosphere formation capacity of glioblastoma stem cells. Figure S4. Effect of glutamine deprivation and replenishment on stemness of cancer cells. Figure S5: Glutamine depletion increases ROS through lowering glutathione level. Figure S6: Morphology change induced by hydrogen peroxide. Figure S7. Effect of glutamine depletion on the expression of β-catenin targeted genes. Figure S8: Effect of glutamine on SP cells. Table S1. Primers used for quantitative real-time PCR assay. Table S2. Primers used for quantitative real-time PCR assay. (PPTX 762 kb) [file 12943_2017_623_MOESM1_ESM.pptx]

## Slide 1
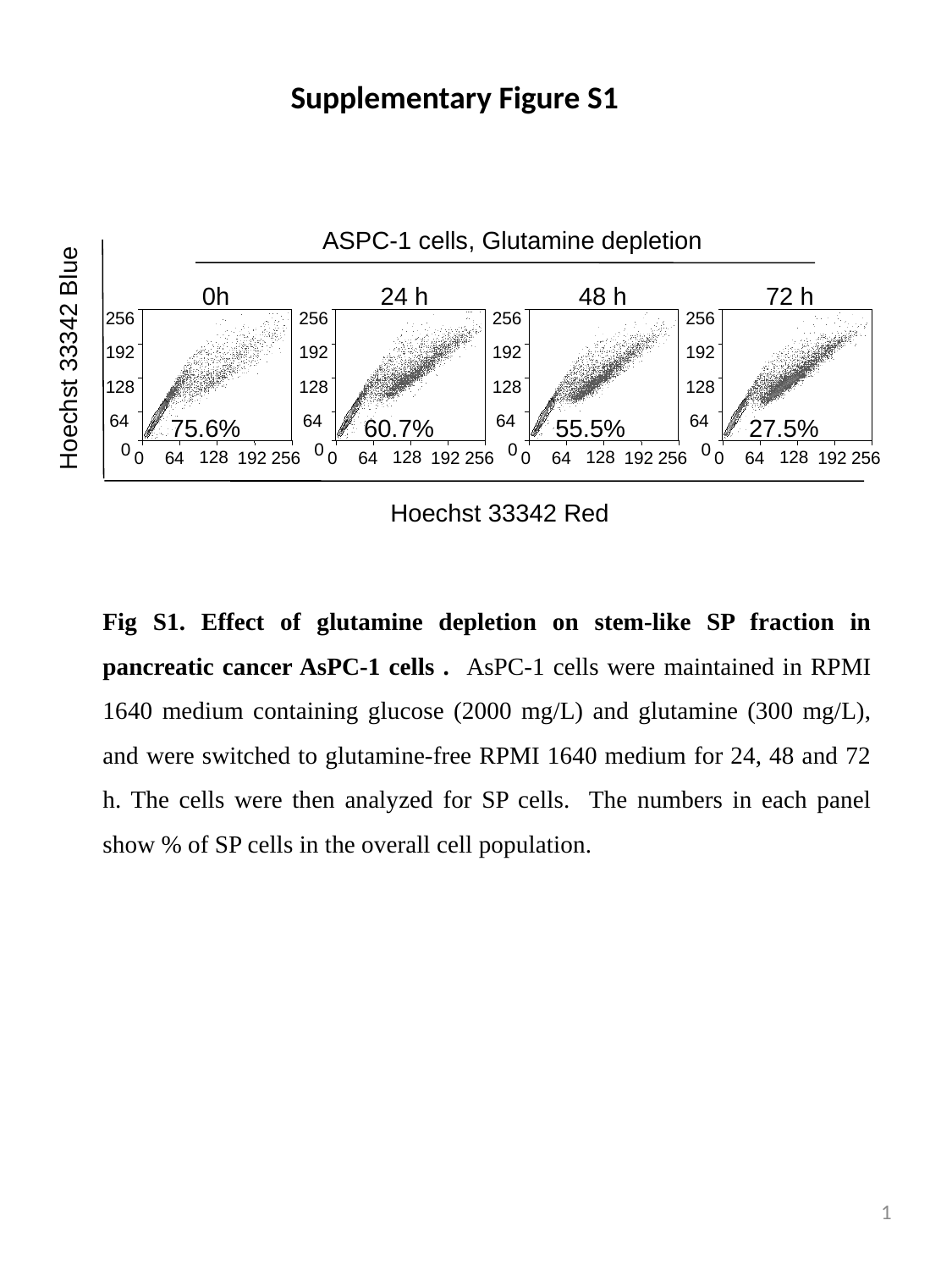

Supplementary Figure S1
Hoechst 33342 Blue
0h
24 h
48 h
72 h
256
192
128
64
0
128
64
192
256
0
75.6%
256
192
128
64
0
128
64
192
256
0
60.7%
256
192
128
64
0
128
64
192
256
0
55.5%
256
192
128
64
0
128
64
192
256
0
27.5%
Hoechst 33342 Red
ASPC-1 cells, Glutamine depletion
Fig S1. Effect of glutamine depletion on stem-like SP fraction in pancreatic cancer AsPC-1 cells . AsPC-1 cells were maintained in RPMI 1640 medium containing glucose (2000 mg/L) and glutamine (300 mg/L), and were switched to glutamine-free RPMI 1640 medium for 24, 48 and 72 h. The cells were then analyzed for SP cells. The numbers in each panel show % of SP cells in the overall cell population.
1

## Slide 2
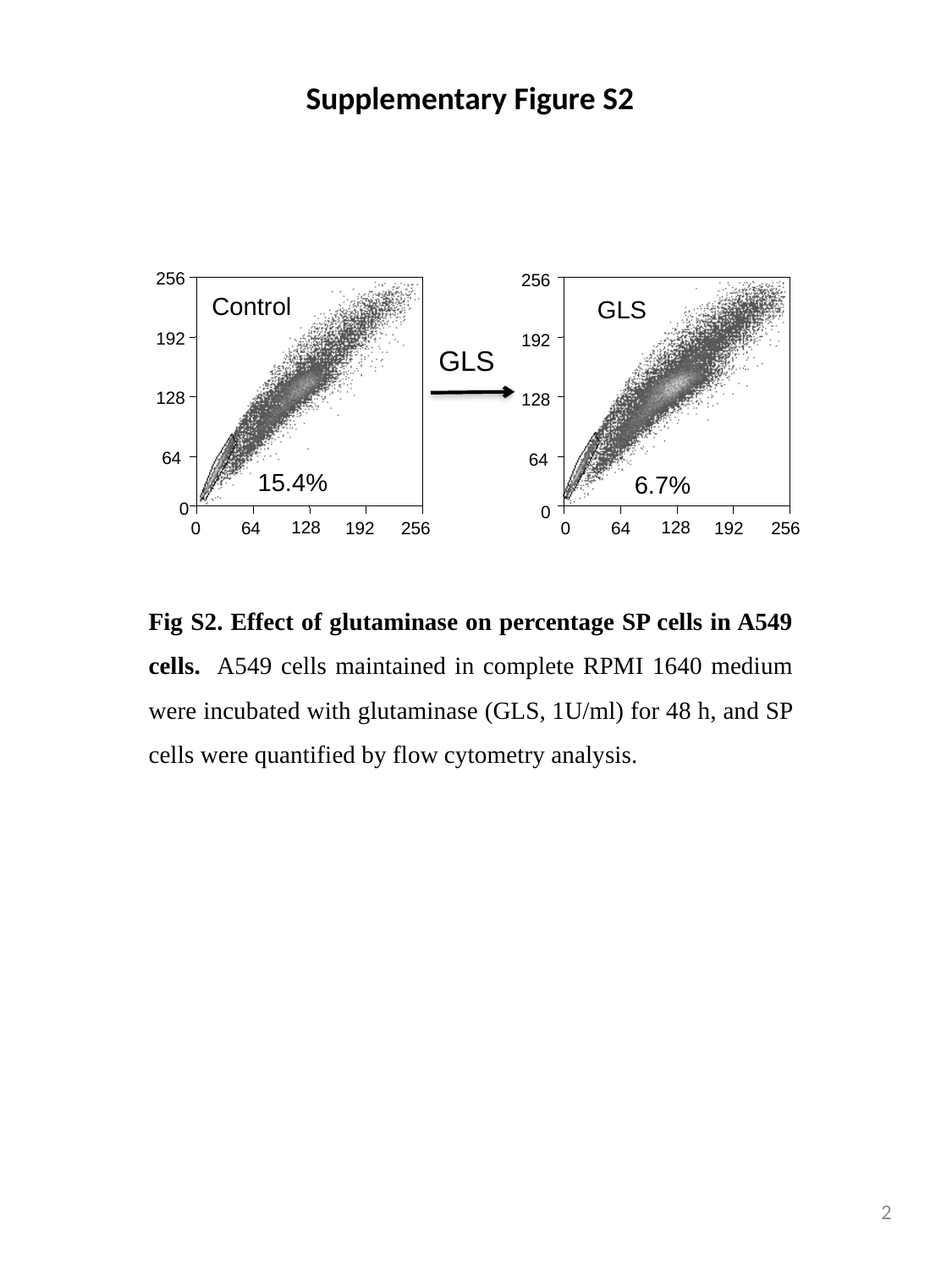

Supplementary Figure S2
256
192
128
64
0
128
0
64
192
256
15.4%
Control
256
192
128
64
0
128
0
64
192
256
6.7%
GLS
GLS
Fig S2. Effect of glutaminase on percentage SP cells in A549 cells. A549 cells maintained in complete RPMI 1640 medium were incubated with glutaminase (GLS, 1U/ml) for 48 h, and SP cells were quantified by flow cytometry analysis.
2

## Slide 3
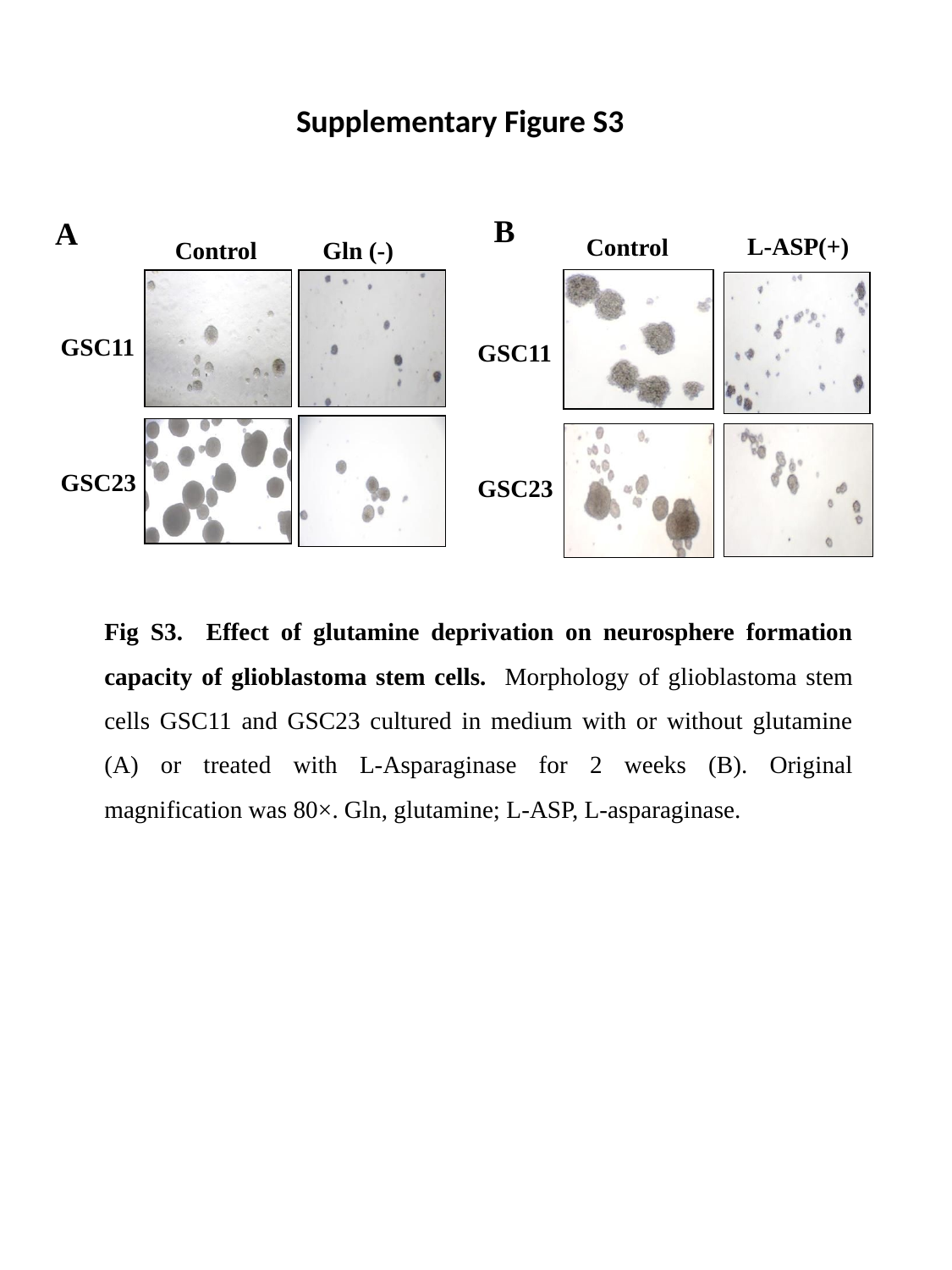

Supplementary Figure S3
B
A
Control
Gln (-)
GSC11
GSC23
L-ASP(+)
Control
GSC11
GSC23
Fig S3. Effect of glutamine deprivation on neurosphere formation capacity of glioblastoma stem cells. Morphology of glioblastoma stem cells GSC11 and GSC23 cultured in medium with or without glutamine (A) or treated with L-Asparaginase for 2 weeks (B). Original magnification was 80×. Gln, glutamine; L-ASP, L-asparaginase.

## Slide 4
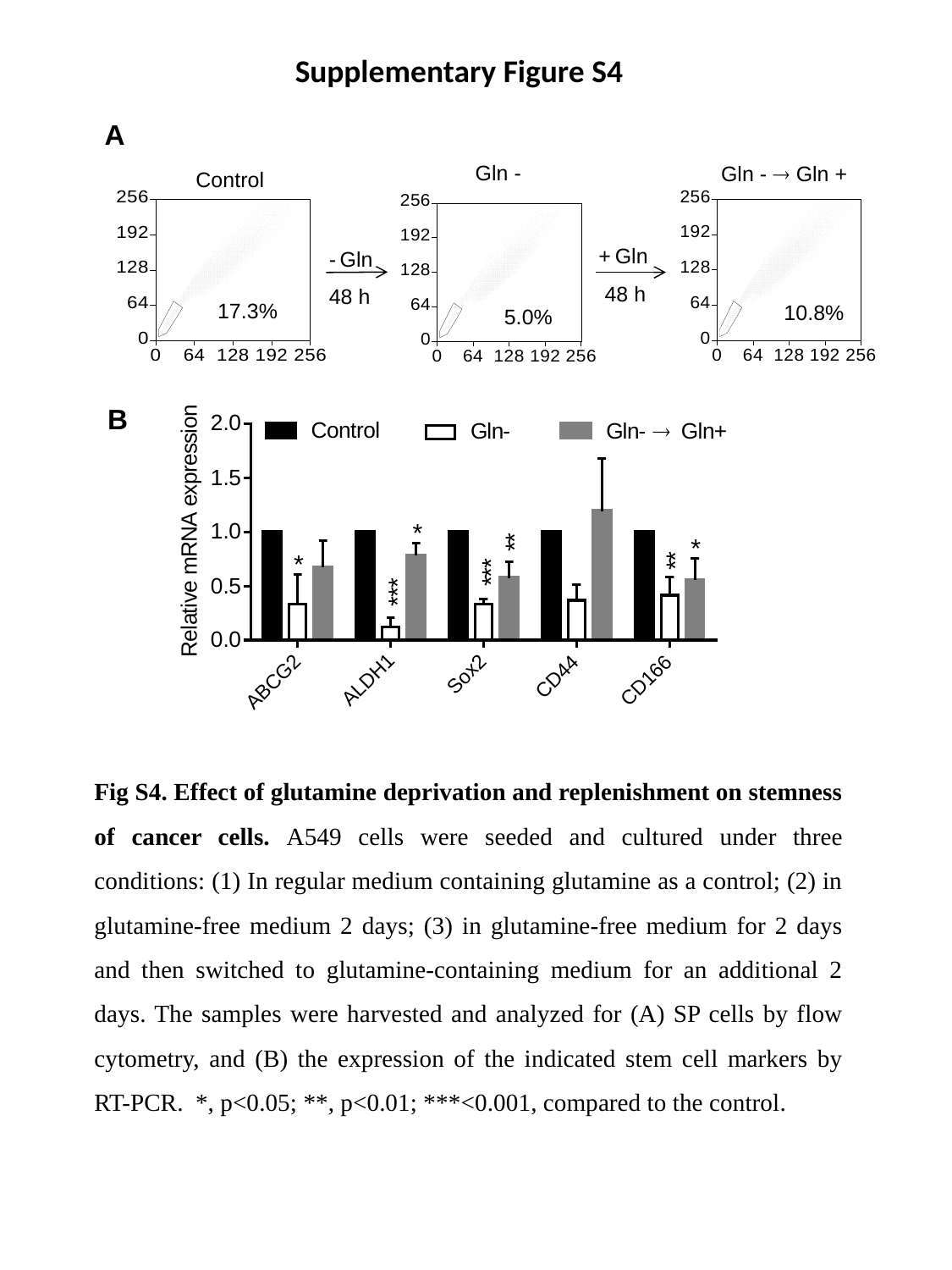

Supplementary Figure S4
A
Gln -
Gln -  Gln +
Control
17.3%
10.8%
5.0%
+ Gln
 48 h
 - Gln
 48 h
B
Fig S4. Effect of glutamine deprivation and replenishment on stemness of cancer cells. A549 cells were seeded and cultured under three conditions: (1) In regular medium containing glutamine as a control; (2) in glutamine-free medium 2 days; (3) in glutamine-free medium for 2 days and then switched to glutamine-containing medium for an additional 2 days. The samples were harvested and analyzed for (A) SP cells by flow cytometry, and (B) the expression of the indicated stem cell markers by RT-PCR. *, p<0.05; **, p<0.01; ***<0.001, compared to the control.

## Slide 5
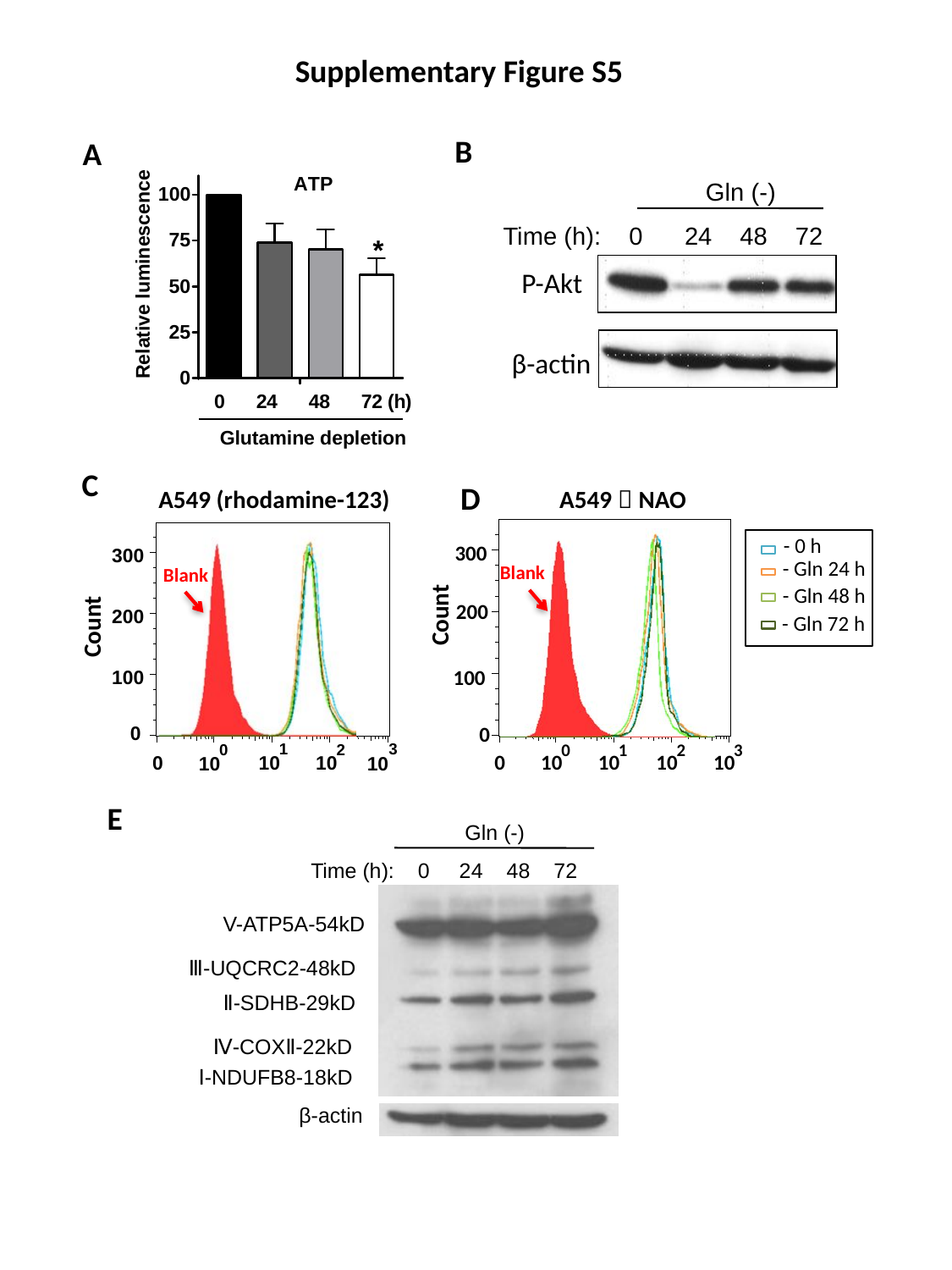

Supplementary Figure S5
B
A
 Gln (-)
Time (h): 0 24 48 72
P-Akt
 β-actin
C
D
A549 (rhodamine-123)
300
200
Count
100
0
1
3
2
0
10
0
10
10
10
Blank
A549（NAO）
300
Count
200
100
0
0
1
2
3
10
10
10
10
0
Blank
 - 0 h
- Gln 24 h
- Gln 48 h
- Gln 72 h
E
 Gln (-)
Time (h): 0 24 48 72
V-ATP5A-54kD
Ⅲ-UQCRC2-48kD
Ⅱ-SDHB-29kD
Ⅳ-COXⅡ-22kD
Ⅰ-NDUFB8-18kD
β-actin
5

## Slide 6
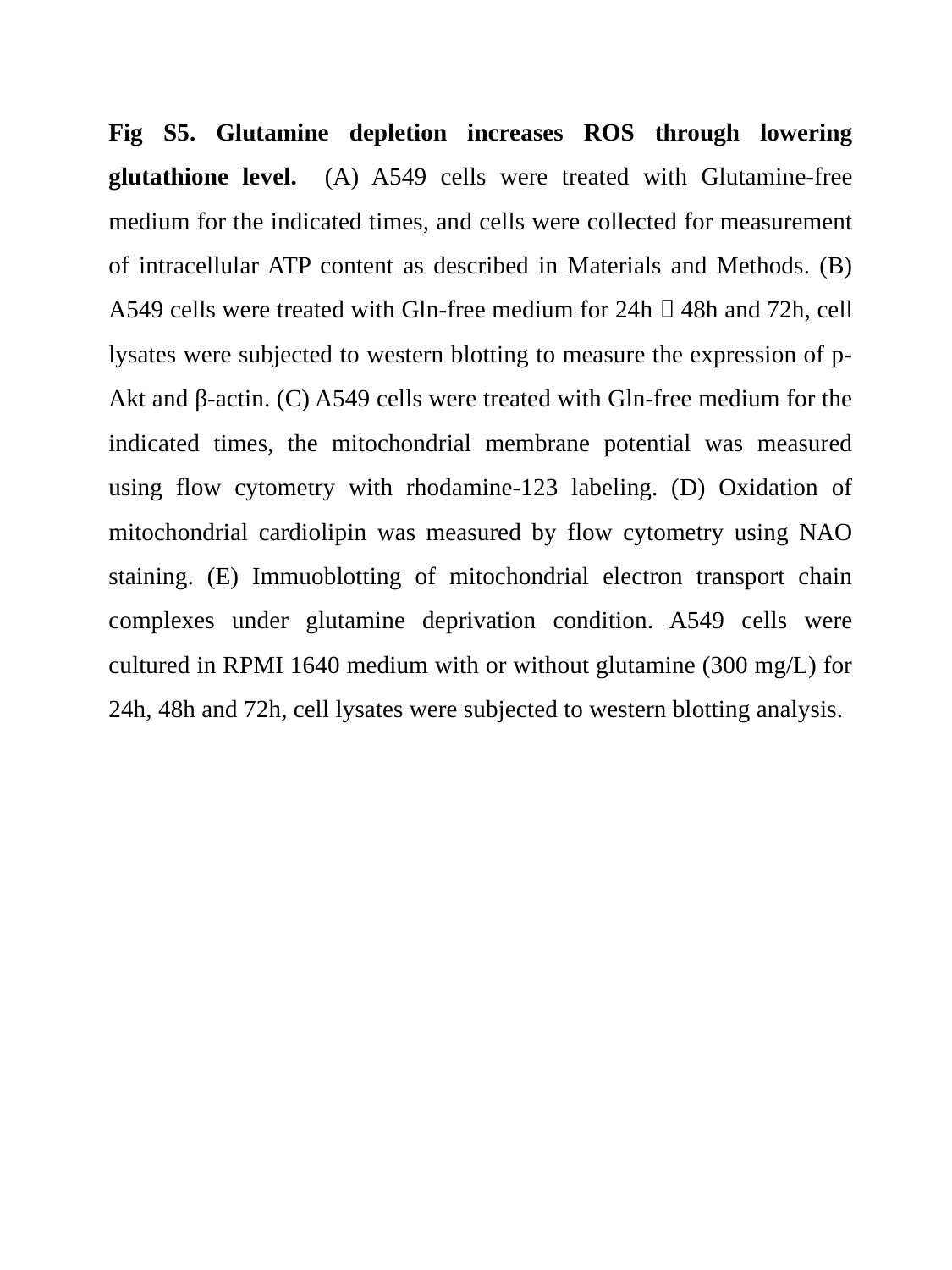

Fig S5. Glutamine depletion increases ROS through lowering glutathione level. (A) A549 cells were treated with Glutamine-free medium for the indicated times, and cells were collected for measurement of intracellular ATP content as described in Materials and Methods. (B) A549 cells were treated with Gln-free medium for 24h，48h and 72h, cell lysates were subjected to western blotting to measure the expression of p-Akt and β-actin. (C) A549 cells were treated with Gln-free medium for the indicated times, the mitochondrial membrane potential was measured using flow cytometry with rhodamine-123 labeling. (D) Oxidation of mitochondrial cardiolipin was measured by flow cytometry using NAO staining. (E) Immuoblotting of mitochondrial electron transport chain complexes under glutamine deprivation condition. A549 cells were cultured in RPMI 1640 medium with or without glutamine (300 mg/L) for 24h, 48h and 72h, cell lysates were subjected to western blotting analysis.

## Slide 7
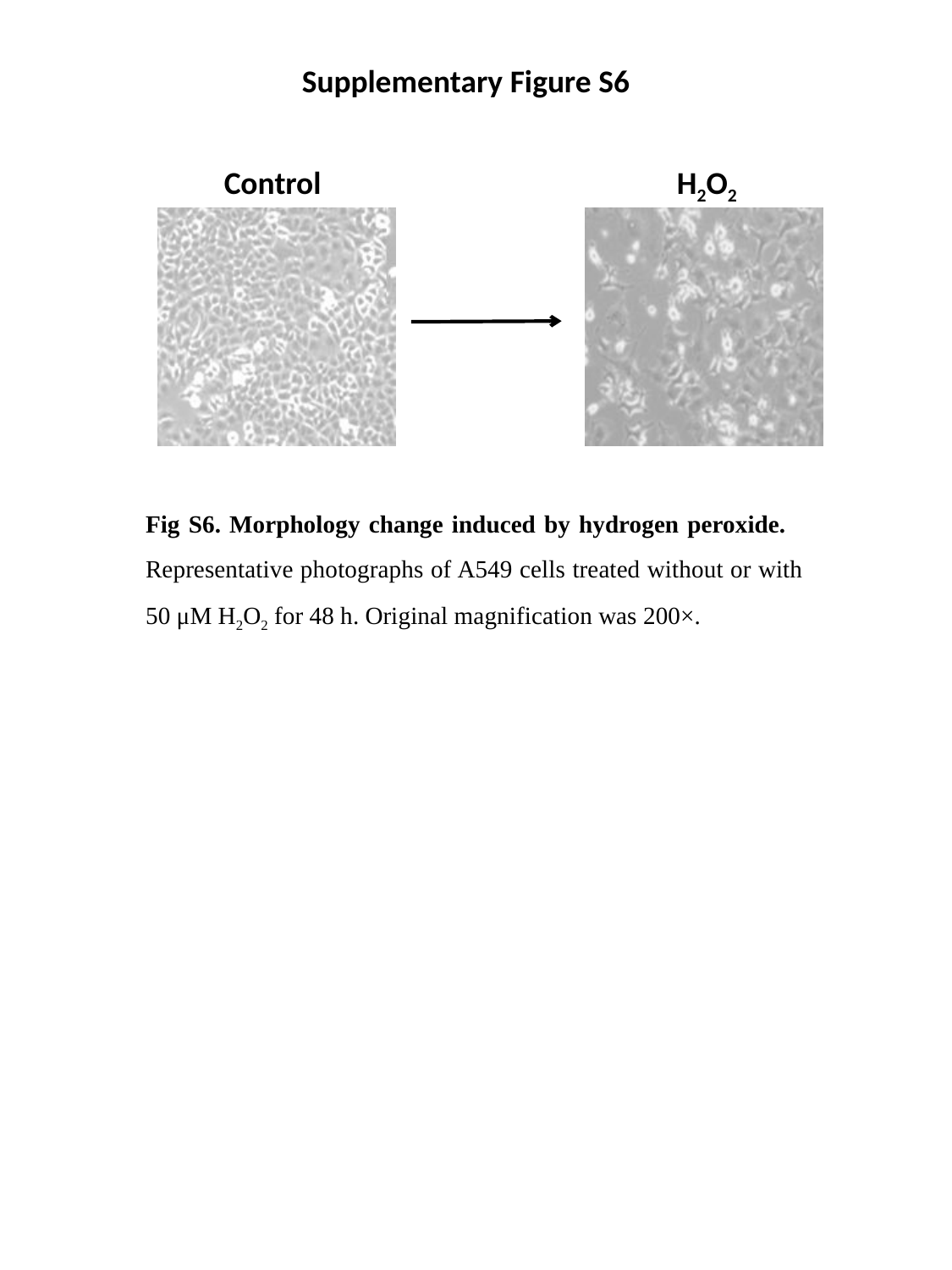

Supplementary Figure S6
Control
H2O2
Fig S6. Morphology change induced by hydrogen peroxide. Representative photographs of A549 cells treated without or with 50 μM H2O2 for 48 h. Original magnification was 200×.

## Slide 8
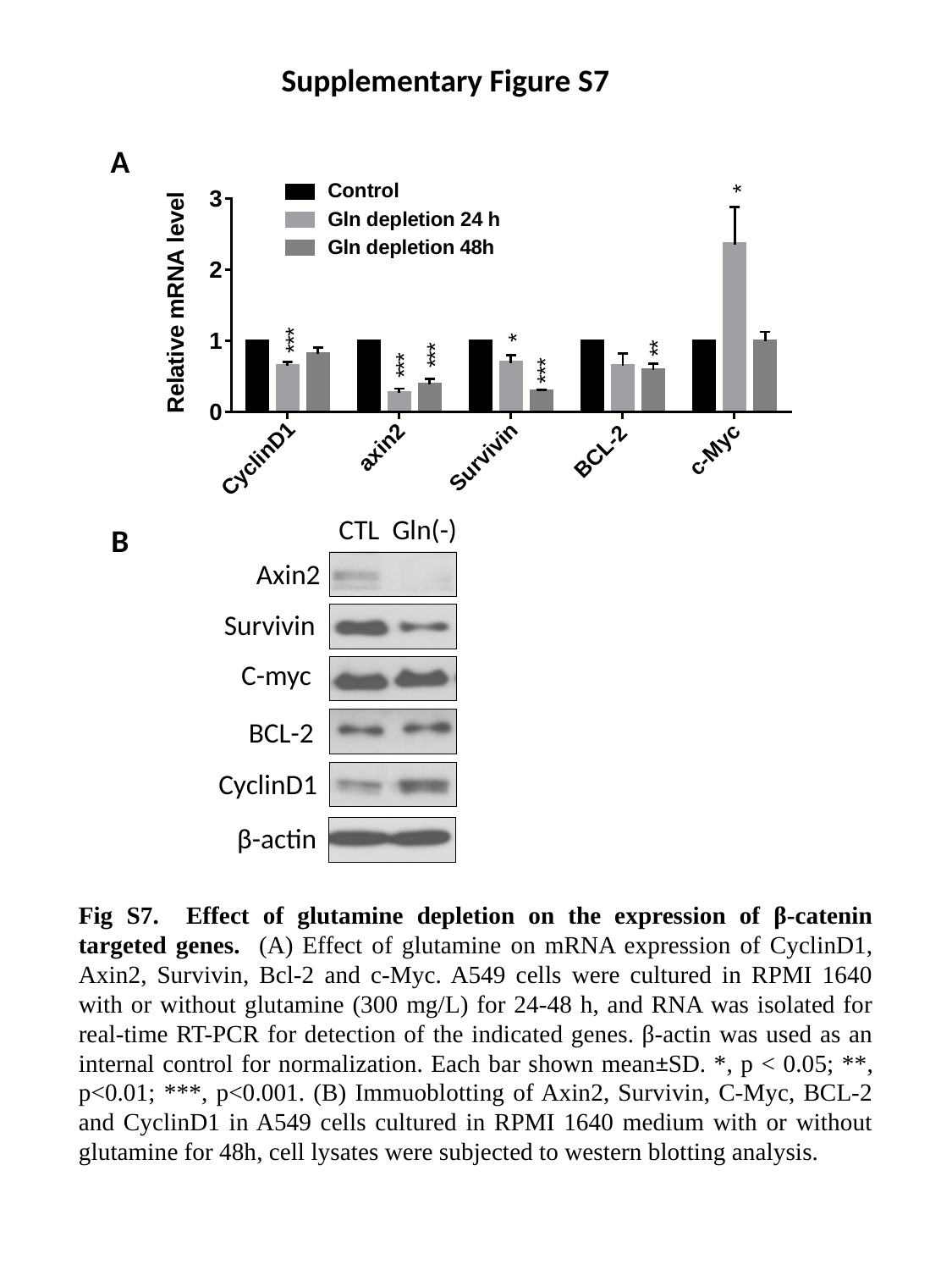

Supplementary Figure S7
A
CTL Gln(-)
Axin2
Survivin
C-myc
BCL-2
CyclinD1
 β-actin
B
Fig S7. Effect of glutamine depletion on the expression of β-catenin targeted genes. (A) Effect of glutamine on mRNA expression of CyclinD1, Axin2, Survivin, Bcl-2 and c-Myc. A549 cells were cultured in RPMI 1640 with or without glutamine (300 mg/L) for 24-48 h, and RNA was isolated for real-time RT-PCR for detection of the indicated genes. β-actin was used as an internal control for normalization. Each bar shown mean±SD. *, p < 0.05; **, p<0.01; ***, p<0.001. (B) Immuoblotting of Axin2, Survivin, C-Myc, BCL-2 and CyclinD1 in A549 cells cultured in RPMI 1640 medium with or without glutamine for 48h, cell lysates were subjected to western blotting analysis.

## Slide 9
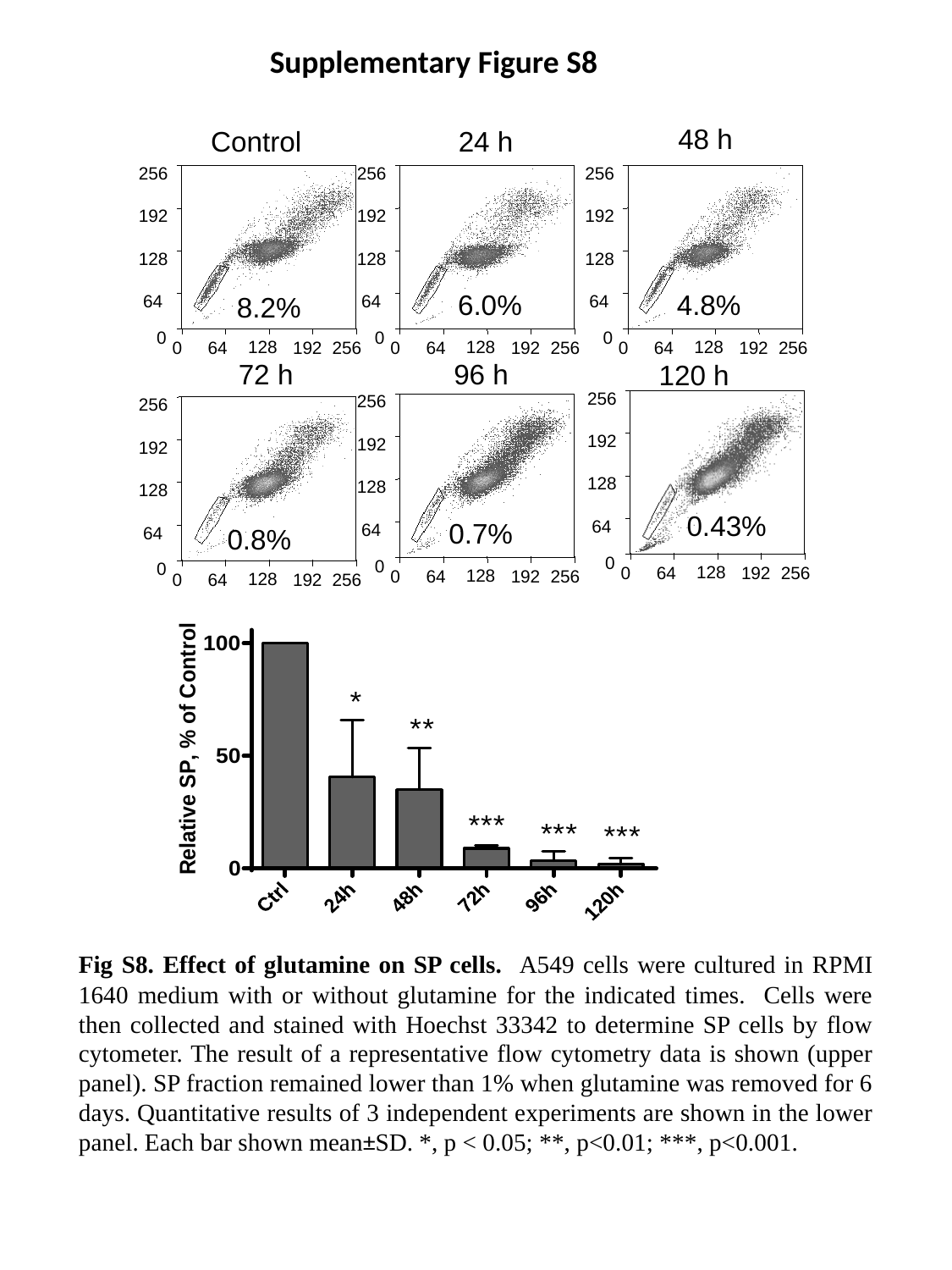

Supplementary Figure S8
48 h
256
192
128
64
0
128
0
64
192
256
4.8%
Control
256
192
128
64
0
128
0
64
192
256
8.2%
24 h
256
192
128
64
0
128
0
64
192
256
6.0%
72 h
256
192
128
64
0
128
0
64
192
256
0.8%
96 h
256
192
128
64
0
128
0
64
192
256
0.7%
120 h
256
192
128
64
0
128
0
64
192
256
0.43%
Fig S8. Effect of glutamine on SP cells. A549 cells were cultured in RPMI 1640 medium with or without glutamine for the indicated times. Cells were then collected and stained with Hoechst 33342 to determine SP cells by flow cytometer. The result of a representative flow cytometry data is shown (upper panel). SP fraction remained lower than 1% when glutamine was removed for 6 days. Quantitative results of 3 independent experiments are shown in the lower panel. Each bar shown mean±SD. *, p < 0.05; **, p<0.01; ***, p<0.001.

## Slide 10
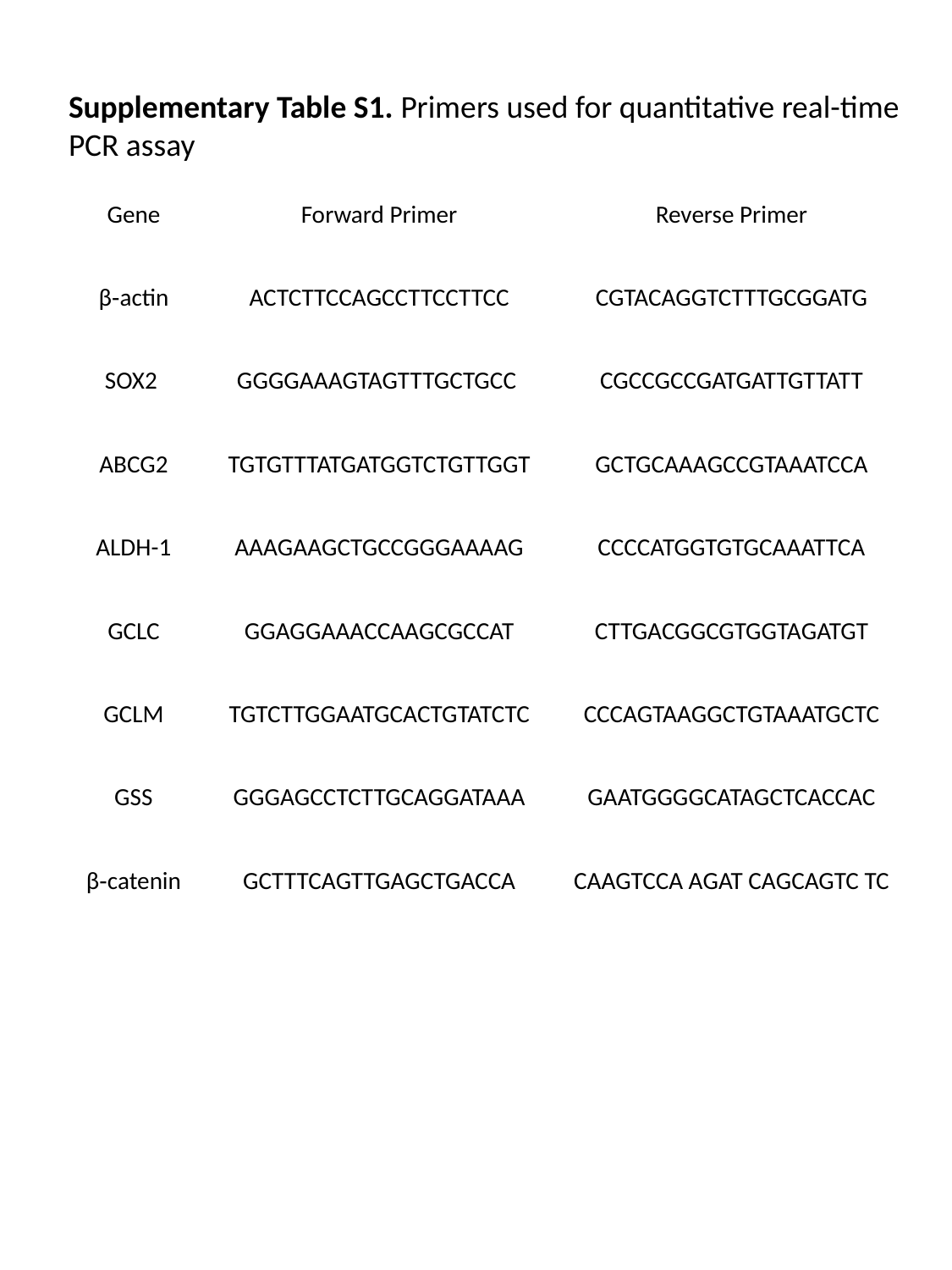

Supplementary Table S1. Primers used for quantitative real-time PCR assay
| Gene | Forward Primer | Reverse Primer |
| --- | --- | --- |
| β‐actin | ACTCTTCCAGCCTTCCTTCC | CGTACAGGTCTTTGCGGATG |
| SOX2 | GGGGAAAGTAGTTTGCTGCC | CGCCGCCGATGATTGTTATT |
| ABCG2 | TGTGTTTATGATGGTCTGTTGGT | GCTGCAAAGCCGTAAATCCA |
| ALDH-1 | AAAGAAGCTGCCGGGAAAAG | CCCCATGGTGTGCAAATTCA |
| GCLC | GGAGGAAACCAAGCGCCAT | CTTGACGGCGTGGTAGATGT |
| GCLM | TGTCTTGGAATGCACTGTATCTC | CCCAGTAAGGCTGTAAATGCTC |
| GSS | GGGAGCCTCTTGCAGGATAAA | GAATGGGGCATAGCTCACCAC |
| β‐catenin | GCTTTCAGTTGAGCTGACCA | CAAGTCCA AGAT CAGCAGTC TC |

## Slide 11
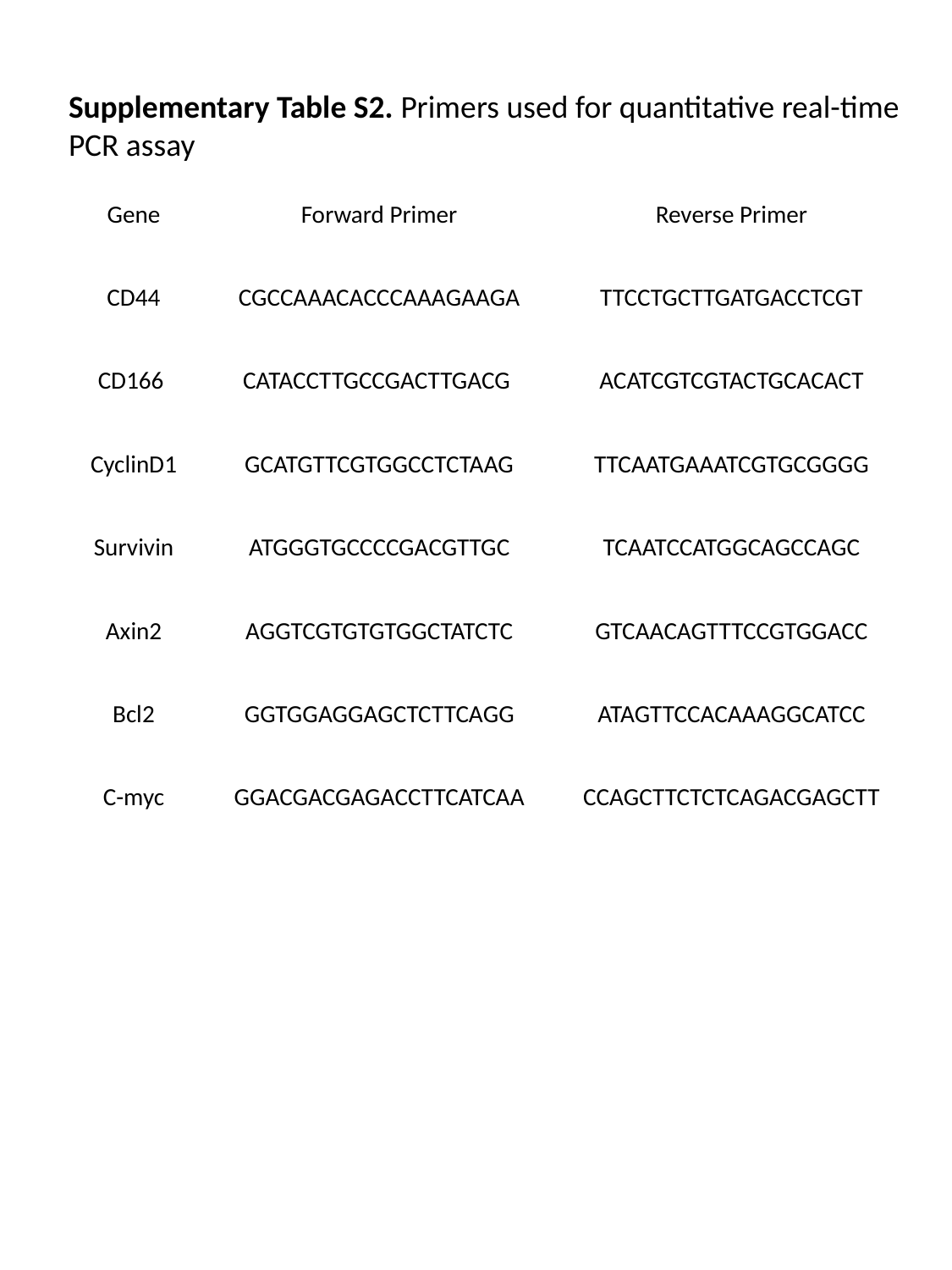

Supplementary Table S2. Primers used for quantitative real-time PCR assay
| Gene | Forward Primer | Reverse Primer |
| --- | --- | --- |
| CD44 | CGCCAAACACCCAAAGAAGA | TTCCTGCTTGATGACCTCGT |
| CD166 | CATACCTTGCCGACTTGACG | ACATCGTCGTACTGCACACT |
| CyclinD1 | GCATGTTCGTGGCCTCTAAG | TTCAATGAAATCGTGCGGGG |
| Survivin | ATGGGTGCCCCGACGTTGC | TCAATCCATGGCAGCCAGC |
| Axin2 | AGGTCGTGTGTGGCTATCTC | GTCAACAGTTTCCGTGGACC |
| Bcl2 | GGTGGAGGAGCTCTTCAGG | ATAGTTCCACAAAGGCATCC |
| C-myc | GGACGACGAGACCTTCATCAA | CCAGCTTCTCTCAGACGAGCTT |
